# Mesopic binocular contrast sensitivity under glare conditions after myopic corneal laser refractive surgery

**DOI:** 10.1007/s00417-025-06882-x

**Published:** 2025-06-28

**Authors:** Lennart M. Hartmann, Franziska Boemer, Anna Hillenmayer, Armin Wolf, Christian M. Wertheimer

**Affiliations:** 1 Department of Ophthalmology, University Hospital Ulm, Prittwitzstraße 43, 89075 Ulm, Germany; 2 Department of Ophthalmology, Ulm University, Prittwitzstraße 43, 89075 Ulm, Germany

**Keywords:** Contrast sensitivity, Glare, Corneal refractive surgery, Corneal higher order aberrations

## Abstract

**Purpose:**

Corneal laser refractive surgery is a common procedure for achieving spectacle independence. Impairment of contrast sensitivity following corneal refractive surgery can have far-reaching consequences, such as restrictions for certain professions or driving bans. The objective of this study was to assess contrast sensitivity following corneal refractive surgery and to identify risk factors contributing to lower contrast sensitivity.

**Methods:**

This retrospective study included 174 eyes with a history of corneal laser refractive surgery for myopia correction ≥ 6 months prior who underwent Scheimpflug imaging, a clinical examination, and a test for mesopic binocular contrast sensitivity in glare mode using a Nyctometer. Potential predisposing risk factors were compared between groups with higher and lower binocular contrast sensitivity.

**Results:**

The uncorrected distance visual acuity was ≥ 0.2 logMAR in 98% of eyes, and the postoperative spherical equivalent was between − 1.5 D and + 1.5 D in 99% of eyes. Moreover, 98% of these patients exhibited a good contrast_(Weber)_ of better than 63%. A statistically significant association was observed between the decentration of the effective optical zone (*p* = 0.01), postoperative UDVA (*p* = 0.01), densitometry (*p* = 0.03) and the maximum myopic meridian (*p* = 0.04) with lower contrast sensitivity.

**Conclusions:**

The overall refractive and contrast sensitivity outcomes of myopic corneal laser refractive surgery are favourable. In our population, both lower uncorrected distance visual acuity and higher decentration of the effective optical zone are correlated with lower contrast sensitivity.

## Abstract (highlights)

**Key messages:**

*****What is known***:**

- ● Corneal laser refractive surgery has a generally favourable refractive outcome.
- ● There is a degree of contention surrounding its impact on contrast sensitivity, especially in the context of occupational medicine.

*****What is new***:**

- ● The present study evaluated a cohort of 174 eyes regarding the postoperative mesopic binocular contrast sensitivity under glare conditions following myopic corneal laser surgery, and potential risk factors associated with lower contrast sensitivity.
- ● Refractive outcome and overall contrast sensitivity outcomes were favourable. However, a statistically significant correlation was identified between lower postoperative uncorrected distance visual acuity and higher decentration of the effective optical zone with lower binocular contrast sensitivity under glare conditions.

## Introduction

Corneal laser refractive surgery, which encompasses procedures such as photorefractive keratectomy (PRK), small incision lenticule extraction (SMILE), laser-assisted subepithelial keratomileusis (LASEK) and in situ keratomileusis (LASIK) is a prevalent and widely accepted surgical procedure for achieving spectacle independence. As the global incidence of ametropia, particularly myopia, continues to rise, this figure is likely to increase in the future [1].

The success of a refractive procedure is typically quantified by assessing the postoperative uncorrected distance visual acuity (UDVA). The uncorrected visual acuity after corneal refractive surgery is typically excellent [2]. However, it is important to note that central visual acuity is only one part of the human visual perception. Another crucial aspect of visual perception is contrast sensitivity, which refers to the capacity to consistently identify a visual target on a standard luminance background [3]. Contrast sensitivity can be defined as the difference in luminance of an object visible against a background of different luminance [4]. A reduction in contrast sensitivity, particularly in the context of glare sensitivity, has also been demonstrated to have a negative impact on quality of life in other eye diseases [5]. In some countries, contrast sensitivity testing is a mandatory requirement for the issuance of various licences and certifications. A driver’s licence and several other professions require the ability to perceive contrast, and deficits in this area can result in restrictions, loss of job, or a ban on night driving [6].

There is a paucity of evidence regarding the impact of corneal refractive surgery on contrast sensitivity, as mixed results have been reported in the literature. While some studies have reported differences regarding contrast sensitivity between patients with a history of corneal refractive surgery and controls [7], others have not found any significant differences [8]. Therefore, the objective of this study was to evaluate the effect of previous corneal refractive surgery on the mesopic contrast vision or twilight vision with glare and to determine factors that predict its occurrence, such as higher order aberrations and decentration of the effective optical zone.

## Methods

### Study design

This is a retrospective study of 174 eyes of 87 patients who underwent binocular corneal laser refractive surgery for myopia correction more than six months ago. All patients were seen at the Department of Ophthalmology between January 2016 to August 2023. This study was approved by the responsible ethics committee (ethical approval ID: 22/24) and conducted in accordance with the tenets of the declaration of Helsinki.

### Inclusion criteria

Patients who had previously undergone corneal laser refractive surgery were seen in our outpatient clinic and were included if they underwent a complete ophthalmic examination, including best corrected and uncorrected distance visual acuity (CDVA and UDVA) and corneal tomography with Scheimpflug imaging (Pentacam, Oculus, Wetzlar, Germany). The Landolt C optotype was employed to ascertain decimal visual acuity which was subsequently converted to logMAR. Binocular contrast sensitivity (BCS) was assessed using a Nyctometer (Rodenstock, Germany) to evaluate the mesopic contrast vision or twilight vision with glare. Only Scheimpflug imaging without indication of error was deemed suitable for this study. Exclusion criteria included a history of repeated surgery and other corneal and ocular comorbidities affecting the Scheimpflug imaging such as keratoconus, keratectasia, history of keratoplasty or radial keratotomy.

### Measurement of binocular contrast sensitivity

The Nyctometer measures contrast sensitivity under challenging conditions for regulatory purposes in vision-dependent, high-responsibility occupations. This is a standardized illumination under mesopic conditions and challenging oncoming glare, such as occurs when driving at night. The Nyctometer was designed to replicate the conditions of nocturnal traffic, with consideration given to the effects of glare. The contrast figure employed is a dark circular sign displayed against a background of a defined luminance of 0.1 cd/m2, with a protruding line (Hartmann nose). The proband is presented with the same figure at varying orientations. Furthermore, the contrast with the background is varied with each figure. Within the context of glare mode, a glare source of 20 feet, a glare angle of 3 degrees, and a corneal illuminance of 0.35 lx are within the preset parameters. In order to differentiate from visual acuity (VA), the figure is presented in a manner that is clearly discernible above the minimum visual acuity (1.0 logMAR).

### Predictive factors for contrast sensitivity

**Fig. 1:**
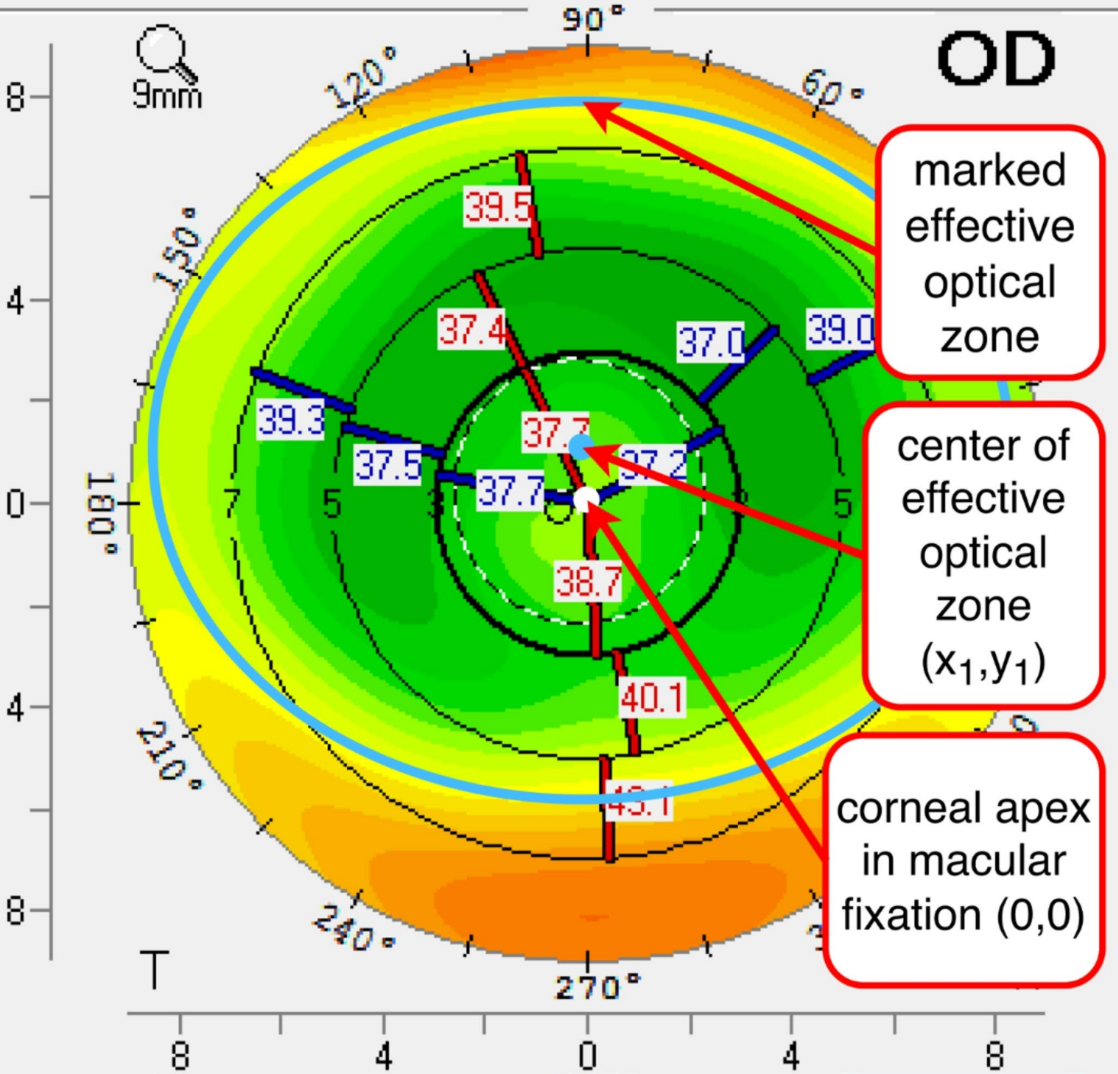
The decentration was determined using the anterior curvature map of Scheimpflug imaging. The location of the ablation profile was estimated by drawing an ellipse into the color-coded map (blue line). The centre of the ellipse (blue dot) was considered to represent the centre of the ablation profile, and the distance between this point and the apex in central fixation (white dot) was calculated using the Pythagorean theorem

Several clinical and imaging parameters were recorded in order to ascertain their impact on contrast sensitivity. Contrast sensitivity was consistently measured binocularly, but the clinical parameters were determined individually for each eye. From the Scheimpflug imaging, the root mean square corneal higher-order aberrations (RMS HOA 4 mm) and the optical densitometry from 0 to 2 mm were collected for each eye. Manifest astigmatism from subjective refraction and uncorrected distance visual acuity were also recorded. The decentered optical effective zone was determined as follows: The ablation profile was estimated by delineating an ellipse on the anterior curvature map within the area exhibiting central corneal flattening subsequent to myopic surgery. Two points were determined as follows: The centre of the ellipse, defined by the coordinates (x1, y1), and the corneal apex as approximation of the fixation, defined by the coordinates (0,0). The distance between the coordinates of the centre of this ellipse and the corneal apex was considered to be the decentration of the effective optical zone. This geometrical distance was calculated using the Pythagorean theorem (Fig. 1).

### Statistical analysis

Excel 365 (Microsoft, Redmond, WA, USA), and GraphPad 10 (GraphPad Software, San Diego, CA, USA) were used for data processing and statistical analysis. Graphs were generated using GraphPad 10. To calculate the effect of the risk factors upon contrast sensitivity, the average value of both eyes were calculated and then the dependency of these average values on binocular contrast sensitivity was estimated [9]. Statistical comparisons were performed employing a linear regression using the method of minimizing the sum of squared error terms. Factors that could influence postoperative binocular contrast sensitivity were identified by exhibiting a slope that was significantly different from zero. A F-test was employed to ascertain statistical significance. A p-value of < 0.05 was considered significant.

## Results

### Patient characteristics

A total of 174 eyes from 87 patients who had previously undergone corneal refractive surgery were included in this study. The mean age of the patients was 23 (± 4) years. Of the total, 35 (40%) patients were female. In each instance, the duration between examination date and surgery date could not be obtained. However, it is important to note that all measurements were conducted as part of an official medical report and in accordance with the established guidelines. A minimum period of six months is required to elapse after surgery before the examinations for the medical report can be issued. Corneal refractive laser surgery procedures included 68 (39%) eyes with history of Femto-LASIK, 78 (45%) cases of LASEK or PRK and 28 (16%) cases of SMILE.

### Refractive outcome

**Fig. 2:**
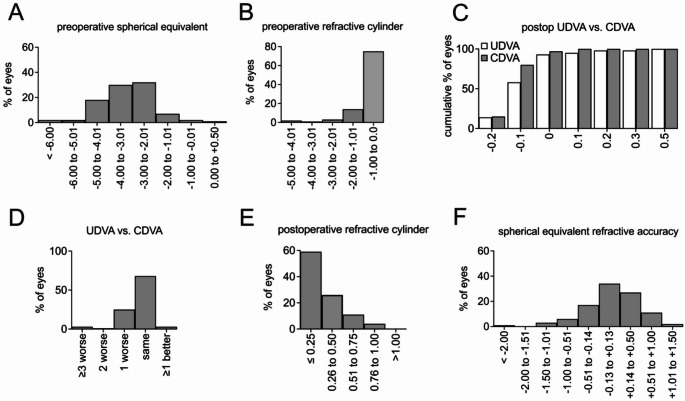
Reports of refractive outcome quality of refractive surgery of our cohort are presented. Preoperative spherical equivalent (**A**) and preoperative refractive cylinder (**B**) are shown. Postoperative spherical equivalent (**C**) and remaining cylinder (**D**) have mostly been within the anticipated target. Postoperative UDVA and CDVA of each eye were plotted (**E**) and directly compared (**F**) to each other

20% of the eyes had a preoperative spherical equivalent lower than − 4 D (Fig. 2A). Additionally, in 89% of the eyes the preoperative cylinder was equal or less than − 2 D (Fig. 2B). The overall refractive outcome of our corneal laser treatment cohort was found to be favourable. The UDVA was better than 0.1 logMAR in 95% of the eyes and better than 0.2 logMAR and 98% of eyes (Fig. 2E). In direct comparison of UDVA and CDVA, only six (3%) eyes exhibited an UDVA that was worse than the CDVA by three Snellen lines or more, while the remainder demonstrated a difference of no more than one line. The target refraction was achieved in majority of cases, with 99% of eyes exhibiting a postoperative spherical equivalent within the range of −1.5 D to + 1.5 D (Fig. 2C). Furthermore, residual refractive astigmatism was minimal, with no patient exceeding one dioptre. Additionally, 85% of the eyes exhibited a postoperative remaining cylinder of equal to or less than 0.5 diopters (Fig. 2D). Furthermore, a low decentration of the effective optical zone was observed, with a mean value of 0.32 (± 0.24) mm. Additionally, low corneal higher-order aberrations (RMS HOA 4 mm) were noted, with a mean value of 0.15 (± 0.06) µm. The densitometry 0–2 mm was also evaluated and showed a mean of 17 (± 2) in the entire cohort.

### Contrast sensitivity

**Fig. 3:**
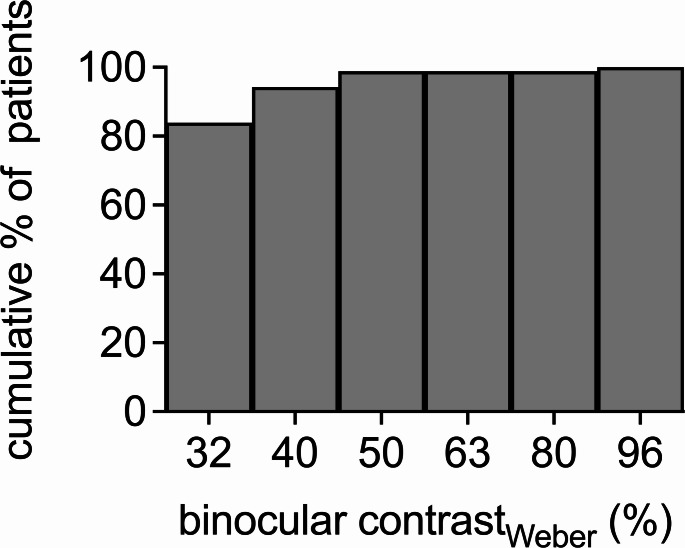
A good contrast sensitivity in mesopic conditions was observed in nearly all patients. Even though some patients achieved better contrast than others, 98% were still within a good range of contrast_(Weber)_ below 63%

In addition to the refractive outcome, the binocular contrast sensitivity in glare mode under mesopic conditions was high in the majority of eyes, with 146 (84%) eyes achieving a very good binocular mesopic contrast_Weber_ in glare mode of 32%. 28 (16%) eyes exhibited a contrast sensitivity inferior to 32% (Fig. 3).

### Difference in contrast sensitivity between different corneal refractive laser surgery procedures

**Fig. 4:**
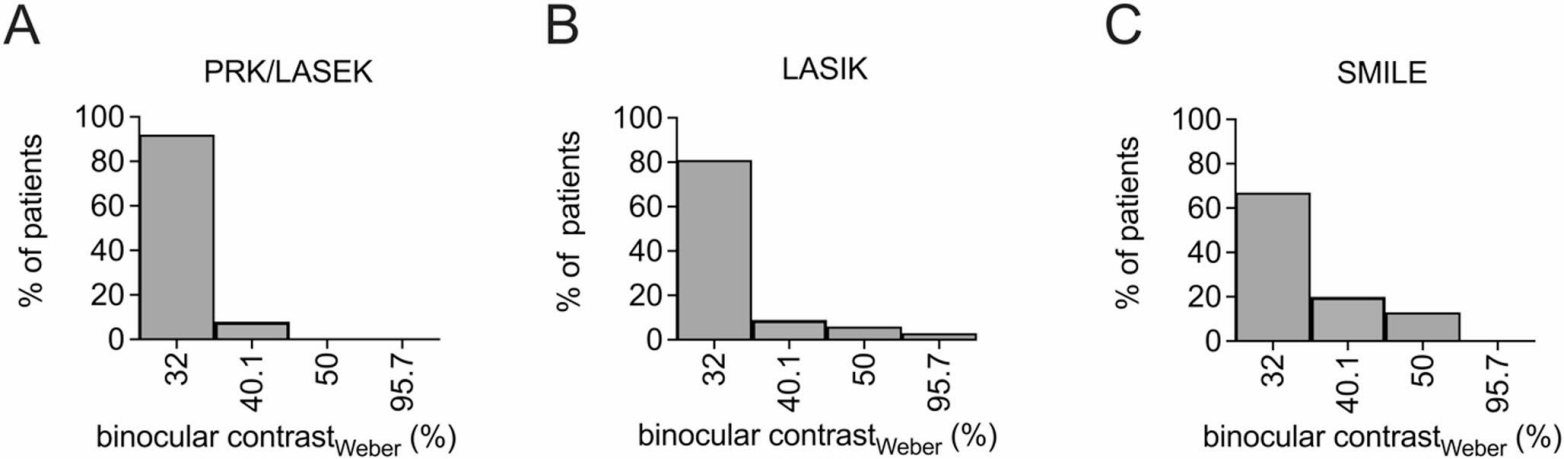
Comparison of postoperative binocular contrast sensitivity after PRK/LASEK (**A**), LASIK (**B**) and SMILE (**C**). A group comparison revealed no statistically significant difference between the different surgical procedures

The binocular contrast sensitivity was generally favourable and comparable in all different corneal ablation methods. A subsequent group comparison between the thre distinct groups revealed no statistically significant difference regarding the postoperative binocular contrast sensitivity between the various refractive laser procedures (Fig. 4).

### Predictive factors for lower contrast sensitivity

**Fig. 5:**
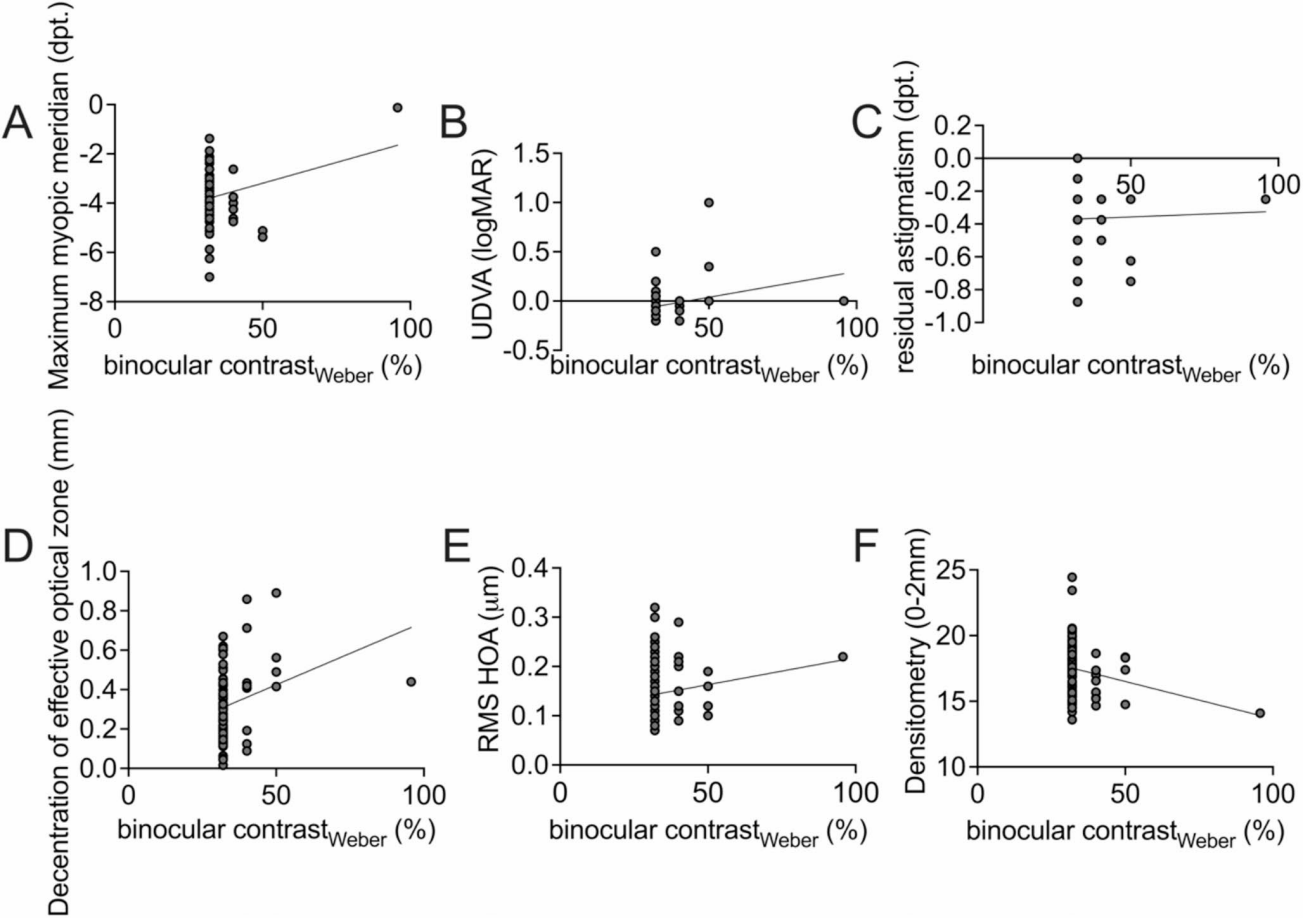
Linear regression of potential predisposing risk factors and binocular contrast sensitivity. The slope between decentration of the effective optical zone (*p* = 0.01), postoperative UDVA (*p* = 0.01), densitometry (*p* = 0.03), maximum myopic meridian (*p* = 0.04) and the binocular contrast sensitivity was significantly different from zero. The slope was not significantly affected by the RMS HOA (*p* = 0.14) and the residual astigmatism (*p* = 0.80)

The potential risk factors were determined by performing a linear regression. Factors that could influence postoperative binocular contrast sensitivity with respect to a slope significantly different from zero, were the decentration of the effective optical zone (*p* = 0.01), postoperative UDVA (*p* = 0.01), densitometry (*p* = 0.03) and the maximum myopic meridian (*p* = 0.04). RMS HOA (*p* = 0.14) and the residual astigmatism (*p* = 0.80) did not significantly affect the slope emphasizing a smaller influence of these factors on binocular contrast sensitivity in our study population (Fig. 5). These results indicate that many factors contribute to postoperative binocular contrast sensitivity after corneal refractive surgery.

## Discussion

Contrast sensitivity is needed to reliably differentiate between a target on a background both with defined luminance [3]. This study, which involved 174 healthy eyes with a history of previous corneal laser refractive surgery that had been performed more than six months previously, demonstrated a favourable refractive outcome, visual acuity and, in particular, normal contrast sensitivity in glare mode [10]. In order to identify the factors responsible for a lower contrast sensitivity, a correlation was established between contrast sensitivity and different postoperative findings. The correlation indicates that both lower uncorrected distance visual acuity and higher decentration of the effective optical zone are associated with lower contrast sensitivity.

The evidence regarding the impact of corneal refractive laser surgery on contrast sensitivity is the subject of considerable debate. It has been demonstrated that corneal refractive surgery tends to impair contrast sensitivity [11, 12]. However, there are also reports that only statistically, but not clinically significant changes can be observed [13], whereas others reported normal contrast sensitivity [14]. Unfortunately, many studies do not explicitly provide a detailed description of normal values for contrast sensitivity. However, some data exists on pilots and train drivers that are often tested for contrast sensitivity deficits in their medical exams [10]. This data is then compared to that of cataract patients in order to determine cut-off values for healthy contrast sensitivity [10]. In our study, we were able to demonstrate mostly normal contrast sensitivity according to these predefined values, with nearly all patients remaining within normal values that allow for normal daily activities and the ability to perform of all professions described in the paper [10].

The contrast sensitivity of an individual is significantly influenced by the ambient light conditions in which they are situated. This can be measured using a variety of methods, with no single gold standard currently in existence [15]. In this study, we have opted for the most challenging conditions of contrast sensitivity in mesopic conditions under the influence of glare. It has been demonstrated that glare at higher illuminance impairs contrast sensitivity in otherwise healthy young participants [15] and the lights of an oncoming car at night can reduce contrast sensitivity by as much as a factor of 6 [16]. A significant number of patients have reported difficulties when driving at night following corneal refractive surgery [17]. Both mesopic and photopic contrast sensitivity can be measured [3, 18]. The role of a loss of the pupillary pinhole effect was achieved as another challenge by choosing mesopic conditions over photopic ones. Notwithstanding, the challenging conditions, our patients exhibited satisfactory contrast sensitivity. The use of additional instruments and a comparison of the observed outcomes could provide further valuable insights, thereby enhancing the comparability of our results to existing literature.

Another critical determinant of visual outcome following any corneal refractive procedure is the effective or functional optical zone [19]. This term refers to the corneal area that is ablated in order to achieve the desired target refraction. Decentration of the aforementioned zone has been demonstrated to have a deleterious effect on visual outcome following corneal refractive surgery [20]. In accordance with this, our study revealed a correlation between decentration of the effective optical zone and a reduction in contrast sensitivity in our study [20]. Furthermore, it is important to acknowledge that a gold standard for determining the effective optical zone has yet to be established [21].

It has been demonstrated that increased corneal higher-order aberrations influence contrast sensitivity following corneal refractive surger [11]. However, our findings did not reveal a correlation between higher-order aberrations and contrast sensitivity. The extent of higher-order aberrations observed in our study was somewhat lower than that reported by others [22]. It is hypothesized that in our study, the inability to detect increased higher-order aberrations, which may explain why contrast sensitivity was not influenced. This is consistent with other literature that did not report an increase in higher-order aberrations after refractive surgery [23]. In contrast, a recent meta-analysis demonstrated that higher-order aberrations were significantly increased [24]. Part of this divergence may be attributed to the differing methodologies employed in the two studies. This discrepancy may be attributed to the relatively strict guidelines set forth by the German Refractive Surgery Committee, which define the depth of ablation and the scope of application in relation to the range of refractive errors treated [25]. Additionally, no hyperopic ablations were included in our study.

Despite the target being presented at 1.0 logMAR and all patients demonstrating superior visual acuity to that of the target, a correlation was identified between contrast sensitivity and uncorrected acuity within our population. This finding is consistent with existing literature indicating that reduced visual acuity with intentionally hyperopic blurring is associated with a reduction in contrast sensitivity [26]. Furthermore, a reduction in contrast sensitivity has been reported for other refractive errors, such as astigmatism [27]. In our study, we observed low residual astigmatism in most of our patients and were unable to reproduce this effect.

This study is limited by its retrospective design and the absence of a control group. The absence of data on certain confounding variables meant that these could not be controlled. Additionally, the decentration of the effective optical zone was marked in a manual manner. Although this process was performed by two different examiners and a third examiner was consulted in cases of disagreement, such a manual task always introduces bias which cannot be avoided. Furthermore, the present study did not investigate changes induced by refractive corneal surgery. In our study, only patients who had previously undergone refractive surgery were included. Consequently, it is not possible to comment on the individual change in contrast sensitivity induced by corneal refractive surgery or to make a comparison between the different surgical techniques used in corneal refractive surgery. Such an investigation would be feasible only in the context of a prospective trial with data collected at both the pre- and postoperative stages.

In conclusion, the results of myopic corneal laser refractive surgery demonstrate an overall positive outcome in terms of refractive and contrast sensitivity. In our population, both lower uncorrected distance visual acuity and higher decentration of the effective optical zone are associated with lower contrast sensitivity .

## Data Availability

The datasets generated during and/or analyzed during the current study are available from the corresponding author on reasonable request.
